# Incidence of cerebral desaturation events in the ICU following cardiac surgery

**DOI:** 10.1186/cc9429

**Published:** 2011-03-11

**Authors:** S Greenberg, A Garcia, L Howard, R Fasanella, J Vender

**Affiliations:** 1 North Shore University Health System, Evanston, IL, USA

## Introduction

We hypothesize that there is a high incidence of cerebral desaturation events (CDE - an absolute decrease in SctO_2 _to <55% for ≥15 seconds) during the first 6 hours of ICU admission following cardiac surgery. Clinical trials have validated transcranial cerebral oximetry, a non-invasive tool that uses near-infrared spectroscopy to measure cerebral oxygen saturation, as a way to detect cerebral ischemia [1]. Cerebral oximetry is frequently used in the intraoperative setting, but rarely utilized postoperatively [2]. We attempted to identify if CDEs occur in the ICU.

## Methods

This IRB-approved, prospective, observational study captures the CDE incidence from 40 ASA IV patients in the ICU period following elective cardiac surgery. Exclusion criteria were: age <18, patients presenting for emergency surgery, and patients undergoing off-pump procedures. The FORE-SIGHT (CAS Medical Systems Inc., Branford, CT, USA) absolute cerebral oximeter monitor remained on patients for the first 6 hours in the ICU. All patients were managed according to the usual ICU standard of care. All care providers were blinded to CDEs during the 6-hour study period. During this time, a portable computer was attached to the cerebral oximeter, bedside physiologic monitor and mechanical ventilator, which recorded all data at 1-minute intervals and allowed data to be stored on a computer database.

## Results

Complete data were collected on 40 high-risk patients (mean age of patients = 71 (36 to 86), mean duration of intubation (hours) = 22.8 (6 to 240), mean duration of ICU stay (days) = 3.3 (1 to 20)). A majority of the patients underwent coronary bypass grafting only or valve only procedures. A high incidence, 13/40 (32.5%), of CDEs was observed in our study cohort, with some episodes exceeding 2 hours. A higher incidence of postoperative nausea/vomiting (PONV) was observed in patients with CDEs (3/13 vs. 0/27).

## Conclusions

This observational trial is the first to demonstrate a high incidence of CDEs in the immediate postoperative period (32.5%) among cardiac surgical patients. Our ongoing observational study will attempt to demonstrate correlations between physiologic parameters and these postoperative CDEs.
